# RMCNet: A Liver Cancer Segmentation Network Based on 3D Multi-Scale Convolution, Attention, and Residual Path

**DOI:** 10.3390/bioengineering11111073

**Published:** 2024-10-27

**Authors:** Zerui Zhang, Jianyun Gao, Shu Li, Hao Wang

**Affiliations:** 1 School of Bioengineering, Chongqing University, Chongqing 400044, China; zhangzerui2218@163.com; 2 Medical Device Institute, Shenyang Pharmaceutical University, Benxi 117004, China; jygao357@gmail.com; 3 Institute for Medical Device Control, National Institutes for Food and Drug Control, Beijing 102629, China

**Keywords:** 3D multi-scale convolution, attention module, residual path, 3D liver cancer segmentation

## Abstract

Abdominal CT images are important clues for diagnosing liver cancer lesions. However, liver cancer presents challenges such as significant differences in tumor size, shape, and location, which can affect segmentation accuracy. To address these challenges, we propose an end-to-end 3D segmentation algorithm, RMCNet. In the shallow encoding part of RMCNet, we incorporated a 3D multiscale convolution (3D-Multiscale Convolution) module to more effectively extract tumors of varying sizes. Moreover, the convolutional block attention module (CBAM) is used in the encoding part to help the model focus on both the shape and location of tumors. Additionally, a residual path is introduced in each encoding layer to further enrich the extracted feature maps. Our method achieved DSC scores of 76.56% and 72.96%, JCC scores of 75.82% and 71.25%, HD values of 11.07 mm and 17.06 mm, and ASD values of 2.54 mm and 10.51 mm on the MICCAI 2017 Liver Tumor Segmentation public dataset and the 3Dircadb-01 public dataset, respectively. Compared to other methods, RMCNet demonstrates superior segmentation performance and stronger generalization capability.

## 1. Introduction

In the field of medicine, liver cancer is a malignant disease that seriously threatens human health. The accurate diagnosis and treatment of early liver cancer are crucial for improving the prognosis of patients. Computed tomography (CT) examination is an important imaging method for the diagnosis of liver cancer [1], which can observe the shape, size, and surrounding tissues of liver tumors and can also observe the blood flow situation of liver tumors through enhanced scanning. Clinical studies have shown that the morphology, size, and degree of enhancement of tumors are closely related to patient survival time [2]. Therefore, performing accurate tumor segmentation on CT images is of crucial importance for the diagnosis, treatment, and survival of liver cancer patients.

Technologies such as computed tomography (CT) and magnetic resonance imaging (MRI) have provided abundant image information for the detection of liver cancer. However, accurately identifying and segmenting the liver cancer area from these massive amounts of imaging data is not an easy task. The liver itself has a complex structure, and the morphology, size, and location of liver cancer are diverse [3]. Generally speaking, the segmentation methods of livers and tumors can be classified into three categories: manual segmentation, semi-automatic segmentation, and automatic segmentation [4]. Semi-automatic segmentation is mainly based on methods such as machine learning [5,6,7]. Although this approach improves time efficiency compared to manual outlining, it still requires human intervention, which can introduce deviations due to human factors. Therefore, research on automated segmentation algorithms that offer both fast segmentation speed and high accuracy becomes particularly important.

Automatic segmentation methods for liver cancer are becoming increasingly popular due to their strong reproducibility and high efficiency. So far, many deep learning models have been introduced [8,9,10,11]. From 2012 to 2015, deep neural networks began to be applied in the tasks of visual recognition [12,13,14]. In 2015, Ronneberger O et al. [15] proposed the Unet structure, and applied Unet to medical pathological images, achieving good segmentation results. Since then, Unet and a series of its variants [16,17,18,19] have been widely proposed and applied to the field of medical image segmentation.

To address the segmentation task of liver tumors, researchers have made significant efforts and conducted extensive studies. Some studies have designed liver cancer segmentation as a two-stage task [20,21,22]. For example, Chlebus G et al. [20] proposed a convolutional neural network approach based on the 2D U-Net architecture and a random forest classifier to segment liver tumors. They first performed liver segmentation to constrain the tumor’s region of interest (ROI), then segmented the liver cancer with the assistance of a random forest classifier, ultimately achieving an average Dice coefficient of 0.65 on the LiTS test set. However, a limitation of their method is that segmentation errors in the liver region can affect the accuracy of liver cancer segmentation. Other studies have also attempted to explore the spatial information between CT image slices during the tumor feature learning process in order to better utilize the three-dimensional information of CT images [11,23]. Li, X. et al. [11] combined FCN with Unet and proposed a H-DenseUNet. H-DenseUNet optimized the feature representations within and between slices and solved the problem that two-dimensional convolution cannot fully utilize three-dimensional spatial information. Apart from convolutional neural networks, the emergence of the Transformer architecture [24] has also provided new approaches for liver tumor segmentation. Xie Y et al. [25] bridged convolutional neural networks and Transformers to address the limitations of long-range dependencies in convolutional networks, achieving precise 3D medical segmentation. Recent studies [26,27,28] have also utilized Transformers as encoders to learn features from 3D medical images, achieving promising performance in many multi-organ segmentation tasks.

In segmentation tasks, the edge information of the target is a key factor influencing the decision-making of neural networks. However, the repeated downsampling during the encoding phase may lead to the loss of detailed information that contains rich edge information. In addition, for the liver cancer segmentation task, liver tumors often have a situation where the tumor size and location differences are relatively large, this makes the diversity of features that the model needs to extract increase. Some models are relatively sensitive to the scale of the segmentation target, and targets with inconsistent sizes may cause adaptability problems in the model. To enhance the feature extraction capability for tumor edge information, Seo et al. [29] proposed an improved UNet, which incorporates residual paths during the encoding phase to reduce information loss caused by downsampling. The study only considered the impact of information loss due to repeated downsampling on liver cancer segmentation, without fully accounting for the effect of the diversity of liver cancer characteristics on the model’s adaptability. According to the diversity of liver cancer characteristics, Wang X et al. [30] designed the CPAD-Net network, which uses the CPAM module to capture the most relevant channel and spatial information from multi-scale data, enabling the fusion of contextual multi-scale features. This network performs well in small tumor segmentation tasks but shows limitations in edge detection for larger tumors.

In this paper, in order to fully utilize the three-dimensional spatial information of CT images; address the segmentation challenges posed by the significant variations in the size, shape, and location of liver cancer; and avoid errors introduced by pre-segmentation of the liver region, we take the five-layer encoding and decoding network as the basic network framework, integrate the 3D multi-scale convolution (3D-Multiscale convolution) module [31] and the convolutional block attention module (CBAM) [32] into the shallow encoding of the network, and combine it with the residual path to propose a new network architecture—RMCNet. The 3D multi-scale convolution in the encoder combines multiple convolution operations with different receptive fields, allowing for the simultaneous extraction of both coarse-grained and fine-grained features of liver cancer. This helps the model capture the overall and local structures of the tumor more comprehensively, reducing the impact of variations in tumor shape, size, and location. The convolutional attention module consists of two components: the channel attention module and the spatial attention module. These modules enable the model to focus more effectively on key features relevant to tumor segmentation during training while reducing interference from irrelevant features, thereby improving segmentation accuracy. Additionally, the residual path assists the model in obtaining high-resolution, high-level features during feature extraction. These features often contain rich contour information about liver cancer, guiding the segmentation model to adjust the representation of local boundary features more accurately. Notably, RMCNet directly segments liver cancer from CT images, avoiding the impact of liver region segmentation errors on liver cancer segmentation. Additionally, as a 3D network, RMCNet fully leverages spatial information, addressing the limitations of 2D networks in capturing 3D spatial details.

In summary, the contributions made by this article are as follows:

(1) We integrated multi-scale convolutions with different receptive fields into the encoding part of the model to enable the extraction of fine-grained features, allowing the model to capture tumor characteristics of various sizes and improve segmentation efficiency.
(2) By utilizing 3D CBAM for spatial feature encoding and channel importance evaluation, the model is guided to focus on the location and shape information of liver cancer during the learning process, thereby improving the overall segmentation performance of the model.
(3) We designed residual paths for each encoder in the network to capture more high-resolution advanced features, guiding the segmentation model to accurately locate the boundaries of liver cancer.

The rest of this paper is organized as follows. Section 2 describes our proposed model, datasets, and experimental details, and Section 3 presents the experimental results and conducts the corresponding analysis. Finally, Section 4 concludes this work.

## 2. Materials and Methods

This section mainly introduces the proposed methods and the materials used in this study. Section 2.1 provides a detailed explanation of the structure and principles of the proposed method. Section 2.2 describes the datasets used and the data preprocessing methods. Section 2.3 presents the evaluation metrics employed to validate the model’s performance, and Section 2.4 outlines the specific experimental details.

### 2.1. Model Structure

This subsection provides a detailed explanation of the method proposed in this study and its underlying principles.

#### 2.1.1. Overview of the Model Structure

In the CT images of liver cancer, significant differences in tumor size, shape, and location present challenges. To address these issues, we incorporated the 3D multi-scale convolution (3D-Multiscale Convolution) module and the convolutional block attention module (CBAM) into the network’s encoding part. By using multi-scale convolution to extract tumor features at different granularities and employing CBAM to better focus on the tumor’s shape and location, we enhance the feature extraction process. Additionally, a residual path is added to each encoding layer to further enrich the extracted feature maps.

**Figure 1:**
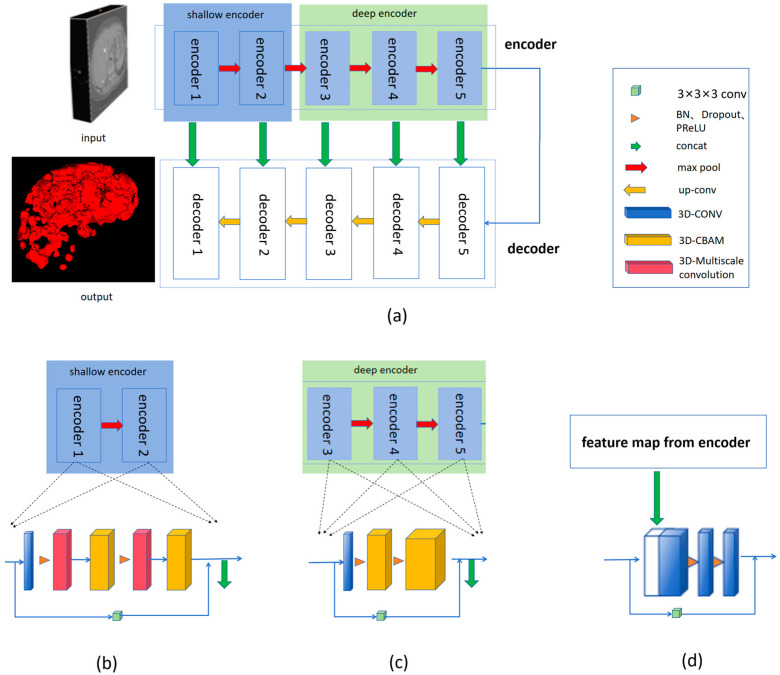
Diagram of the liver cancer segmentation model. Each three-dimensional CT image input is divided into patches of size 96 × 96 × 64 and fed into the model. The encoding stage of the model is divided into the shallow encoding stage and the deep encoding stage. By integrating 3D multi-scale convolution, attention modules, and residual paths to learn features, accurate tumor segmentation is achieved. (**a**) The overall design diagram of the RMCNet model structure; (**b**) the design of the model’s shallow encoder; (**c**) the design of the model’s deep encoder; (**d**) the design of the model’s decoder.

The structural diagram of the model proposed in this article is shown in Figure 1. RMCNet is a 3D convolutional neural network that effectively utilizes three-dimensional spatial information throughout the layer-by-layer convolution process. The 3D-Multiscale Convolution module integrates convolution operations with multiple receptive fields, thereby enriching the features extracted by the model. The CBAM consists of two sub-modules: channel attention and spatial attention, which help the model focus on feature channels and spatial information relevant to liver cancer segmentation during training. The residual path compensates for the lack of long-term dependencies in convolution operations by extracting high-resolution features, enhancing the representation of liver cancer edge information, and improving the model’s ability to locate liver cancer boundaries. This capability is one of the key factors affecting the performance of segmentation models. In the encoder section of RMCNet, we integrate the 3D-Multiscale Convolution module and the CBAM into the shallow encoder. As shown in Figure 1b, the shallow encoder consists of two identical sub-encoders, each containing one convolution layer, two 3D-Multiscale Convolution modules, and two CBAMs. The structure of the deep encoder differs from that of the shallow encoder; as illustrated in Figure 1c, the deep encoder comprises three identical sub-encoders, each containing one convolution layer and two CBAMs. To assist the model in extracting high-resolution features, we also designed a residual path for each shallow and deep sub-encoder to enhance the model’s ability to locate liver cancer boundaries. The decoder of RMCNet consists of five identical sub-decoders, each containing three convolution layers and a residual path. Additionally, we adopted skip connections from the UNet architecture in the decoder section to fully fuse shallow and deep features, further enhancing the model’s ability to recognize liver cancer boundaries.

To ensure that the model has greater stability, generalization ability, and expressive power, as shown in Figure 1, we sequentially introduced Batch Normalization (BN), Dropout, and Parametric ReLU (PReLU) between each convolutional layer and module. Batch Normalization (BN) standardizes each mini-batch of data, reducing internal covariate shift within the network, thereby improving training stability and accelerating model convergence. Dropout, as a regularization technique, randomly drops neurons to effectively reduce model complexity and mitigate the risk of overfitting. Parametric ReLU (PReLU) is an improved version of the Rectified Linear Unit (ReLU). ReLU is a commonly used activation function that applies a nonlinear transformation by setting all negative values to zero while keeping the positive values unchanged, thus introducing nonlinearity into the network. Parametric ReLU (PReLU), as an improved version of ReLU, introduces a learnable parameter to control the slope of the negative region, thereby enhancing the model’s nonlinear expressive ability.

#### 2.1.2. Residual Path

**Figure 2:**
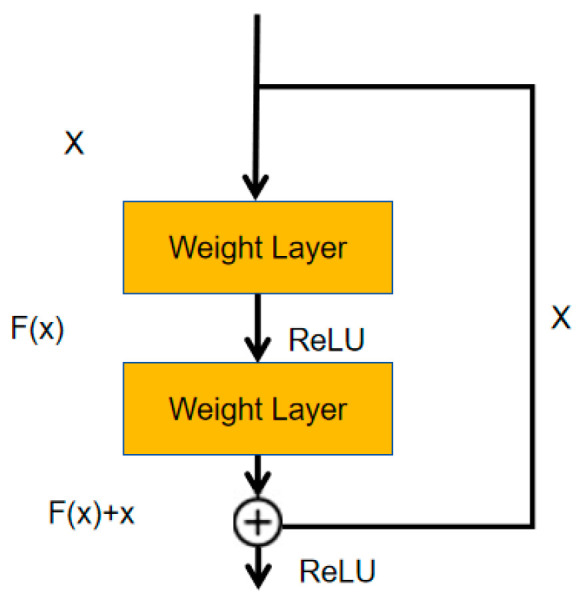
The structure of the residual block.

Convolutional network models usually utilize increased network layers and channel numbers to enhance the feature mining ability of the model. However, excessive network depth and width will lead to higher training errors, and the classification performance of the model will decline. The residual block effectively alleviates the vanishing gradient caused by the increase in parameters in the network [33]. As shown in Figure 2, the residual block introduces a short-circuit connection; that is, the input feature can directly reach the output layer. The residual can be calculated through the input vector and the expected result. As in Equation (1), $x_{l}$ represents the input vector of the lth layer, and $H(x_{l})$ is the expected output.

(1) $$F(x_{l})=H(x_{l})-x_{l}$$

**Figure 3:**
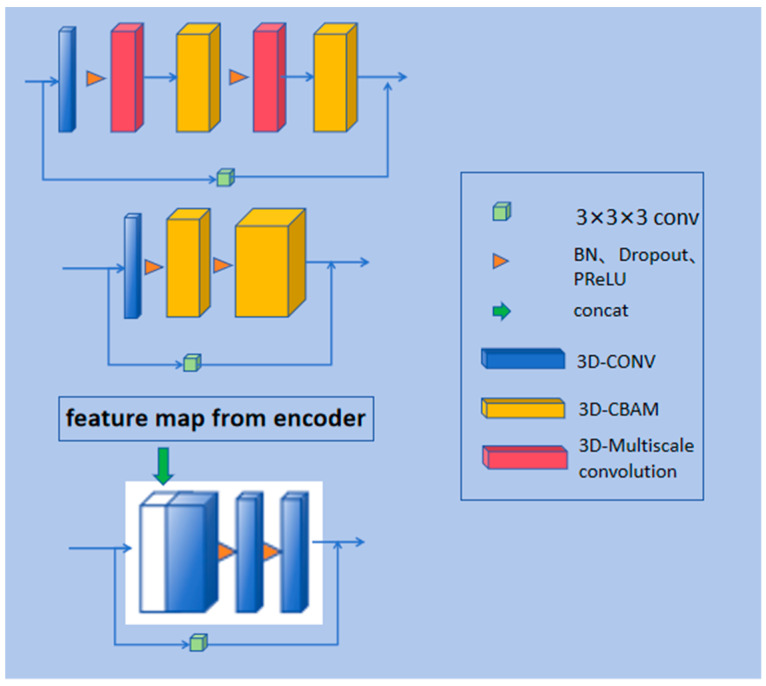
The design of residual path.

In both the encoding and decoding stages of the model, this paper introduces residual blocks in each encoder and decoder layer to prevent issues of gradient vanishing and gradient explosion during the training process and to facilitate better model training. The specific structure of the residual path is shown in Figure 3. The model proposed in this paper draws inspiration from the design of residual blocks and incorporates convolutional layers into the residual connections to form residual paths, thereby extracting more features from the liver cancer regions. Ultimately, the extracted features are added to the features extracted by the encoder itself, effectively achieving the extraction of high-resolution edge information of the segmentation target.

#### 2.1.3. Multi-Scale 3D Convolution

**Figure 4:**
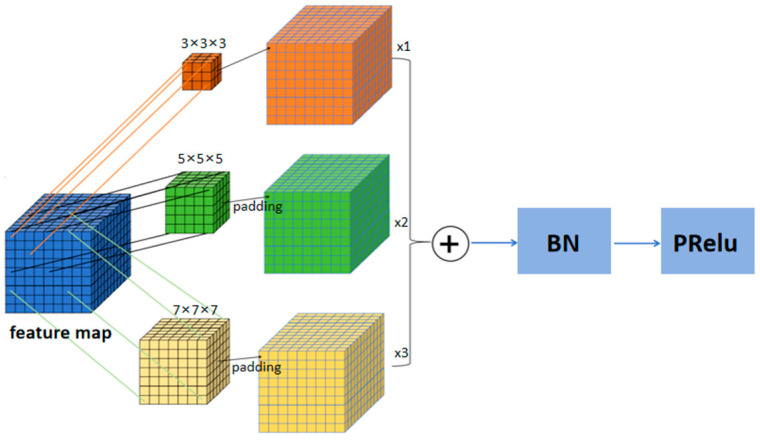
The design of 3D multi-scale convolutional module.

Multi-scale 3D convolution is a type of convolution operation that captures features of varying scales in three-dimensional space. In this paper, multi-scale 3D convolution [31] is incorporated into the shallow U-Net architecture. By combining multi-scale 3D convolution with convolution operations across multiple receptive fields, the features extracted by the model are enriched. Figure 4 illustrates the multi-scale 3D convolution structure presented in this paper, featuring convolution kernel sizes of 3 × 3 × 3, 5 × 5 × 5, and 7 × 7 × 7. In the segmentation tasks discussed, the 3 × 3 × 3 convolution focuses on local areas of the input image, primarily extracting fine texture and edge information of the liver tumor. In the LiTS dataset, liver and tumor tissues often exhibit different texture characteristics; thus, fine texture information is crucial for distinguishing between these tissues. Additionally, edge information aids in determining the extent and shape of the tumor, ensuring the accuracy and integrity of the segmentation results. In comparison, the 5 × 5 × 5 convolution has a larger receptive field, covering a broader local area and extracting features that contain more contextual information. This is beneficial for recognizing relationships between the tumor and surrounding liver tissues, and for capturing shape and texture features of larger areas. The 7 × 7 × 7 convolution, being the largest kernel, captures a wider range of regional features and extensive contextual information. Through this kernel, the overall shape of the tumor and its structural features across a larger range can be captured. Together, the three convolution kernels form the multi-scale convolution feature extraction module. Their cooperative operation enables the model to perform comprehensive feature extraction on both liver and tumor tissues from different angles and scales. The 3 × 3 × 3 kernel provides detailed local features, the 5 × 5 × 5 kernel serves as a transitional scale, and the 7 × 7 × 7 kernel enriches feature representation from a macroscopic perspective. This synergy enhances the model’s ability to tackle the segmentation challenges posed by significant variations in tumor shapes and sizes, thereby improving segmentation accuracy and reliability.

The specific structure of the 3D multi-scale convolution module is shown in Figure 4, where convolution kernels of sizes 3 × 3 × 3, 5 × 5 × 5, and 7 × 7 × 7 extract features from the input feature maps. The features extracted by these different-sized kernels are then fused, allowing the model to better manage the significant variations in tumor shapes and sizes. To ensure that the output feature maps maintain consistent dimensions when extracting features with the 5 × 5 × 5 and 7 × 7 × 7 convolution kernels, padding is applied. This facilitates effective fusion of features extracted by kernels of varying sizes along the spatial dimensions.

#### 2.1.4. Convolutional Block Attention Module

The convolutional block attention module (CBAM), as a lightweight and versatile module, extracts feature maps along two dimensions: channels and spatial information. By multiplying the attention weights with the input features, CBAM performs adaptive feature refinement, producing enhanced output features [32].

**Figure 5:**
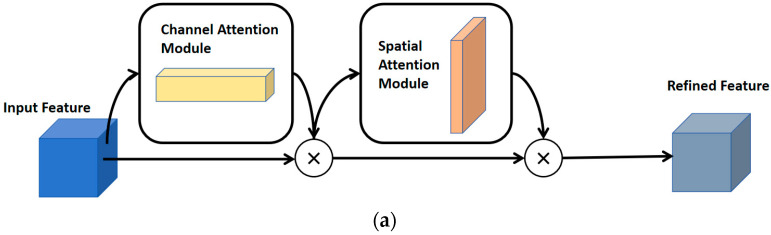

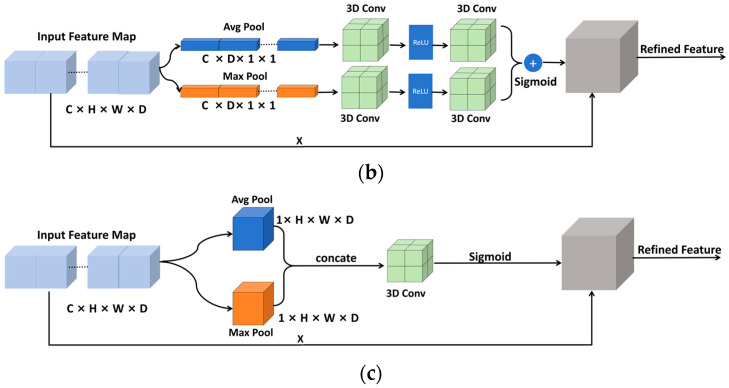
Schematic diagram of the Convolutional Attention Module (CBAM). The CBAM consists of a Spatial Attention Module (SAM) and a Channel Attention Module (CAM). (**a**) The overall structure diagram of the Convolutional Attention Module (CBAM); (**b**) the structure diagram of the Channel Attention Module (CAM); (**c**) the structure diagram of the Spatial Attention Module (SAM).

As shown in Figure 5, this paper extends the general 2D convolutional attention module into 3D. Figure 5a illustrates the overall structure of the 3D-CBAM, while Figure 5b,c depict the respective CAM and SAM sub-modules. As shown in Figure 5a, the CBAM takes the feature map from the previous layer as input, and by passing through the CAM and SAM submodules, it enables dual refinement of the input features. This allows the model to focus simultaneously on meaningful channels and spatial locations, thereby enhancing its feature representation ability and improving decision accuracy. Specifically, the Channel Attention Module (CAM) compresses the input features along the spatial dimensions. First, the input features are processed through average pooling and max pooling operations along the spatial axis, resulting in feature maps with a shape of C × D × 1 × 1. These two feature maps are then passed through a convolutional layer with shared weights to enable channel interaction. The output mappings are combined along the spatial axis to obtain enhanced channel features. Activated by the sigmoid function, after generating the channel weight Mc, a product operation is performed with the original input to obtain the channel attention output feature.

The output calculation formula of the channel attention module is as shown in Equation (2):

(2) $$M_{C}(F)=\sigma (Conv(\gamma (Conv(AvgPool(F))))+Conv(\gamma (Conv(MaxPool(F)))))$$

Among them, F is the input feature map, AvgPool and MaxPool, respectively, represent the global average pooling and the maximum pooling operation, Conv represents the convolution operation, γ represents the ReLU activation function, and σ represents the Sigmoid activation function.

The Spatial Attention Module (SAM) compresses the input features along the channel dimension. It applies average pooling and max pooling operations along the channel axis, resulting in two spatial feature maps with a shape of 1 × H × W × D. These two spatial features are then concatenated along the channel dimension. The output map is reduced in the channel dimension by 3D convolution to be consistent with the original input channel and then activated by the sigmoid function to obtain the spatial weight Ms. Afterwards, the weight is multiplied by the original input feature to obtain the output of the spatial attention module. The output calculation formula of the spatial attention module is as shown in Equation (3):

(3) $$M_{S}(F)=\sigma (Conv(AvgPool(F);MaxPool(F)))$$

Among them, F is the input feature map, AvgPool and MaxPool, respectively, represent the global average pooling and the maximum pooling operation, Conv represents the convolutional operation, $\left(AvgPool\left(F\right);MaxPool\left(F\right)\right),$ indicating to concatenate the results of average pooling and maximum pooling along the channel axis, and σ represents the Sigmoid activation function.

The model proposed in this paper embeds the 3D-CBAM into the encoding part of the model to solve the problem of large differences in position and shape in liver cancer data. The channel attention sub-module in the 3D-CBAM can evaluate and emphasize the importance of different channel features, so that the model can focus more on those feature channels related to liver cancer. For example, some channels may contain more key information such as tumor boundaries and internal textures. Through the effect of channel attention, the features of these important channels will be expressed more significantly. The spatial attention sub-module in the 3D-CBAM can highlight important local areas in the three-dimensional spatial dimension. For liver cancer segmentation, the features of the tumor area and its surroundings are unique in the spatial distribution, and 3D-CBAM can guide the model to pay more attention to these key spatial positions, thereby enhancing the extraction ability of features such as tumor shape and position.

#### 2.1.5. Loss Function

The DICE loss function is a commonly used evaluation index and loss function, which is particularly suitable for medical image segmentation tasks. The DICE loss function is based on the DICE similarity coefficient (DSC). The DICE similarity coefficient is an index for measuring the similarity between two sets and is widely used to evaluate the performance of the segmentation model. The formula of the DICE similarity coefficient is as shown in Equation (4):

(4) $$DSC=\frac{2|A\cap B|}{\left|A|+|B\right|}$$

where A is the predicted binary segmentation result (predicted pixel set). B is the actual binary segmentation result (true pixel set). |A∩B| represents the number of pixels in the intersection of the predicted and actual segmentation results. |A| and |B|, respectively, represent the number of pixels of the predicted and actual segmentation results. The DICELoss function is calculated as shown in Equation (5):

(5) DICELoss=1−DSC

The DICELoss function performs excellently when dealing with medical image segmentation tasks. Its advantage lies in dealing with the problem of class imbalance and directly optimizing the model’s segmentation performance. Therefore, we choose the DICELoss function.

### 2.2. Dataset and Data Preparation

The dataset adopted in this paper is the public dataset of liver and liver tumor segmentation obtained from the Liver Tumor Segmentation Challenge (LiTS-ISBI 2017) [34]. The dataset comes from 131 abdominal computed tomography scans. The image size is 512 × 512, and the number of slices varies from 75 to 987. The size and number of liver cancer lesions in the dataset are not uniform. In the CT image, the number of connected domains represents the number of lesions. Therefore, we count the number of lesions contained in each CT image by calculating the connected domains of the label. In order to calculate the volume of each lesion in the CT image, we count the number of voxels occupied by each lesion. The product of the number of voxels and the voxel size is the volume of the lesion, and the voxel size can be obtained through the voxel spacing in each direction of the CT image. In order to exclude the influence of misoperation by the annotators and noise on the model learning, we removed annotated results that are less than 3 voxels as part of the data cleaning process. The lesion sizes after data cleaning range from 1.4622 mm^3^ to 968,616.6677 mm^3^, and the quartiles are 144.92185 mm^3^, 567.0 mm^3^ and 2768.0958 mm^3^, respectively, and the standard deviation is 52,981.1819 mm^3^. The number of lesions contained in each CT image in the dataset is different. Among them, there are CT images of 12 normal patients without liver cancer lesions. There are 71 patients with the number of CT image lesions ranging from 1 to 5, 40 patients with the number of CT image lesions ranging from 6 to 20, and 8 patients with more than 20 lesions. In addition, due to the individual differences in patients and the diffusivity of liver tumors, there are also problems of large differences in shape and location among liver cancer lesions.

We randomly select the data of 80 patients in the dataset for training, the data of 20 patients for tuning, and the data of 31 patients for testing. For the proposed method, first confirm whether the organization form of the data is the channel-first mode, that is, (C, H, W, D) (where C represents the number of channels, H represents the height of the image, W represents the width of the image, and D represents the depth), to ensure that the format of all input data is consistent.

Additionally, to verify the generalization capability of the model, we tested the proposed method using the publicly available 3Dircadb-01 dataset [35]. The 3Dircadb-01 dataset contains 3D CT scan data from 10 female and 10 male subjects, with a slice size of 512 × 512, and the number of slices ranges from 74 to 260.

According to the HU value, different tissue types in the CT image can be distinguished and the lesion area can be identified. Given that the tumor grows on the liver tissue, the surrounding bones, air, or unrelated tissues are likely to interfere with the segmentation result. Therefore, we set the HU window value within the range of −53 to 163 to excise the organs and tissues unrelated to liver cancer segmentation [36].

**Figure 6:**
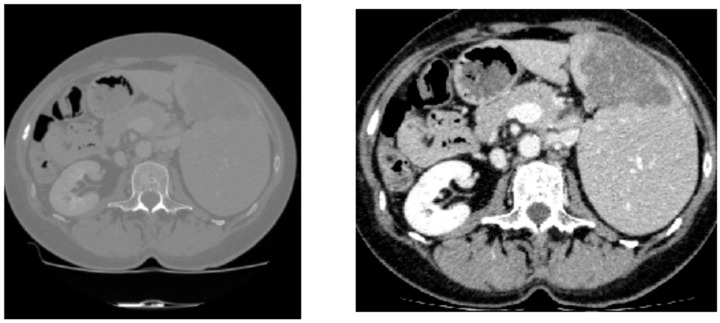
The original image (**left**) and the preprocessed image (**right**).

As shown in Figure 6, in the image preprocessing part, in addition to the conventional HU value truncation, we also use preprocessing operations such as image ROI cropping and data augmentation. ROI cropping includes foreground cropping and liver region cropping. Foreground cropping is cropped according to the size and position of the image foreground frame to remove the background in the image; liver region cropping finds the start slice position and end slice position of the liver region according to the label of the liver in the dataset for slicing processing. The number of slices after processing varies from 50 to 353. The slices in the abdominal CT image that are related to the liver region are selected for model training to make the model more focused on learning the areas related to liver cancer segmentation and improve the goodness of fit of the model. In addition, in order to enhance the diversity of the training data and reduce the risk of model overfitting, we perform random rotation data augmentation on the input images of each batch and randomly rotate the images and labels by 90 degrees with a probability of 50%. These steps ensure that the data are fully preprocessed and enhanced before being input into the model, thereby improving the effect of model training.

### 2.3. Evaluation Metrics

The Dice similarity coefficient (Dice Similarity Coefficient, DSC) is used to measure the similarity between two sets, and the value range is [0, 1]. The larger the DSC value, the more similar the two sets are. It is often used in the task of image segmentation to measure the degree of overlap between the predicted segmentation result and the label to evaluate the accuracy of the segmentation result. The closer its value is to 1, the higher the coincidence degree between the segmentation result predicted by the algorithm and the label, and the better the segmentation effect. Formula (6) is used to calculate DSC. Among them, X represents the predicted result of the segmentation model, and Y represents the label. It can capture the global similarity of the segmentation result, not just the local differences. For unbalanced data, DSC has relatively good tolerance for regions of different sizes.

(6) $$DSC(X,Y)=\frac{2\left|X\cap Y\right|}{\left|X\right|+\left|Y\right|}$$

The Hausdorff distance (Hausdorff Distance, HD) is an index used to evaluate the effect of medical image segmentation, mainly used to measure the maximum distance between two point sets. In the image segmentation task, the contour or boundary of the segmentation result can be regarded as a point set, the contour or boundary of the real target can be regarded as another point set, and then the HD between these two point sets is calculated to reflect the shape and position differences between the segmentation result and the real target. The smaller the HD, the smaller the shape and position differences between the segmentation result and the real target, the better the segmentation effect. Formula (7) is used to calculate HD. Among them, X and Y, respectively, represent the target area predicted by the segmentation model and the target area corresponding to the label. $d_{H}(X,Y)$ represents the bidirectional Hausdorff distance, which has the advantage of being insensitive to noise and outliers. d(x,y) represents the distance between any two points in the two regions X and Y.

(7) dH(X,Y)=maxd(x,y)x∈X y∈Ymax min,d(x,y)y∈Y x∈Xmax min

The Average Surface Distance (Average Surface Distance, ASD) is an index often used to evaluate the effect of medical image segmentation. It measures the accuracy of segmentation by calculating the average distance between the surface point set of the segmentation result and the surface point set of the real target. That is, the surface of the segmentation result is regarded as a point set, the surface of the real target is regarded as another point set, and then the average value of the distances of all corresponding point pairs between these two point sets is obtained. Formula (8) is used to calculate ASD, where A represents the segmentation result, $S_{A}$ represents a certain voxel in the segmentation result, S(A) represents the surface voxel of the segmentation result A, B represents the gold standard, $S_{B}$ represents a certain voxel in the segmentation result, and S(B) represents the surface voxel of the gold standard B. The smaller the ASD value, the closer the segmentation result is to the real target in terms of surface distance, and the better the segmentation effect.

(8) $$ASD(A,B)=\frac{1}{\left|S(A)\right|+\left|S(B)\right|}(\sum_{S_{A}\in S(A)}d(S_{A},S(B))+\sum_{S_{B}\in S(B)}d(S_{B},S(A)))$$

Among them, d(v, S(X)) is defined as the minimum Euclidean distance from voxel v to the surface voxel of the segmentation result X:

(9) $$d(S_{B},S(A))=\underset{S_{x}\in S(X)}{\min}\left\|v-S_{x}\right\|$$

It has certain advantages for evaluating subtle segmentation differences.

The Jaccard coefficient (Jaccard coefficient, JCC) is an index used to measure the similarity between two sets. In image processing, it can be used to measure the degree of overlap between two regions. DSC is a volumetric metric index used to measure the similarity and coincidence degree of the segmentation result in terms of volume. ASD is a metric index based on surface distance used to measure the distance between the surface voxel of the segmentation result and the surface voxel of the gold standard, and it has certain advantages for evaluating subtle segmentation differences. The Jaccard coefficient is used to measure the degree of overlap between the segmentation result and the real target, while HD is used to measure the shape and position differences between the segmentation result and the real target. Using DSC, ASD, JCC, and HD simultaneously to comprehensively evaluate the performance of the segmentation algorithm can help to more comprehensively understand the performance of the proposed algorithm. In order to verify the segmentation performance of the proposed method, we used four indicators of DSC, ASD, JCC, and HD to conduct a comprehensive and complete comprehensive evaluation. This method was compared with the Unet [15], Unetr [26], Vnet [37], Unet3+ [38], MS-FANet [39], and SBCNet [40] methods on the test set, and all the models were trained on the same training set and tuning set.

### 2.4. Experimental Details

During the training process, we set the training rounds of the model to 3000, and conduct verification every two rounds. The learning rate is 0.0001 and the batch size is 2 to ensure that the model can fully learn the features of the data. In order to improve the generalization ability of the model, we also adopt random rotation for data augmentation. DiceLoss function is selected as loss function, and the optimizer is Adam. The kernel sizes of the convolutional layer and the deconvolutional layer in the residual and skip connection are 3 × 3 × 3, respectively, and the stride is 2. The basic hyperparameters of the corresponding network of U-Net are consistent with the original U-Net structure [15]. All the computations in this experiment are carried out on the ubuntu operating system, using two A100 (40 GB memory for each GPU) GPUs. The common deep learning framework pytorch is used as the network architecture and is trained in parallel in the CUDA 11.7 environment [32]. The proposed method was compared with the Unet [15], Unetr [26], Vnet [37], Unet3+ [38], MS-FANet [39], and SBCNet [40] on the test set, and all the models were trained on the same training set.

## 3. Result and Discussion

### 3.1. Parameters and FLOPs

Table 1 shows the parameters, FLOPs, training time, and inference time required for each round of training of the other methods and RMCNet networks. Among them, all models are trained on the same training set and tuning set, and the training parameters are all the same. As the basic model, Unet has a relatively low number of parameters of 1.981 M, the time required for one round of training is 18.10 s, the inference time is 0.542 s, and the FLOPs is 5.239 G, which has good simplicity and efficiency. Vnet was originally proposed for three-dimensional medical images. In the network structure, the short-circuit connection method of ResNet is adopted, and the structure is more complex. The model parameter is as high as 45.598 M, which is about twenty times that of the Unet network, and the time required for one round of training is also longer, the FLOPs is 213.633 G, and the computing resources required for computing are higher. Unet3+ refers to the two network structures of UNet and UNet++, and uses full-scale skip connections and deep supervisions. The model parameter is 22.403 M, which is about ten times that of the Unet network. The MS-FANet model has 10.330 M parameters, which is more than Unet but fewer compared to Vnet and Unet3+. It achieves 43.198 G FLOPs. The training time per round is 30.11 s, and the inference time is 4.873 s. Although the model incorporates residual attention blocks and multi-scale downsampling mechanisms, its efficient feature extraction ensures a relatively balanced performance in terms of computational overhead. The SBCNet model has 9.140 M parameters, with a training time of 41.77 s per round, 39.716 G FLOPs, and an inference time of 4.460 s. While the training time is slightly longer than MS-FANet, SBCNet maintains a lower computational burden. Unetr has 92.618 M parameters and 55.024 G FLOPs. While RMCNet integrates multi-scale convolution, the attention module, and the residual path on the basic encoding and decoding convolutional network framework, the model is more complex and brings more computational overhead, but there is a certain optimization in training efficiency (the time of single-round training of the model is 21.45 s, and the FLOPs increases to 20.397 G).

### 3.2. Results

In order to verify the segmentation performance of the proposed method, we carried out a comprehensive and complete comprehensive evaluation. This method was compared with the Unet [15], Unetr [26], Vnet [37], Unet3+ [38], MS-FANet [39], and SBCNet [40] methods on the same test set, all the models were trained on the same training set and tuning set, and the training parameters were all consistent. Four image segmentation evaluation indicators, including DSC (Dice Similarity Cofficient), HD (Hausdorff Distance), ASD (Average Surface Distance), and JCC (Jaccard coefficient), were used to verify the effectiveness of the proposed method on the test set, and the test results are shown in Table 2. The HD of the RMCNet proposed in this article is 11.07 mm, which is smaller than those of the selected baseline models except for Unetr, and the model has relatively high accuracy and fineness. The error of the model on the surface distance is small, the ASD of 2.54 mm is lower than those of the selected baseline models except for Unetr, and it is relatively accurate in capturing the target boundary. The DSC value is high, and there is a certain guarantee for the segmentation accuracy of the target area by this model. The comprehensive performance of the model is good after combining multiple modules, and the JCC value is the largest compared to other models. Adding a residual path improves the model’s ability to transfer features, making the model learning more effective; the convolutional attention module makes the model more focused on key features and improves the segmentation performance; the 3D multi-scale convolution enhances the model’s processing ability of features of different scales.

To compare the performance differences between the RMCNet and other models, we conducted *t*-test analyses on the four metrics to evaluate the significance of differences between RMCNet and other models for each metric. The statistical results are shown in Table 3: except for the *p*-values greater than 0.05 for the HD and ASD metrics between Unetr and RMCNet, the *p*-values for other models across various metrics are all less than 0.05. The *t*-test analysis allows us to conclude that RMCNet significantly outperforms Unet, Vnet, Unet3+, MS-FANet, and SBCNet in the segmentation performance across the four metrics: DSC, JCC, HD, and ASD. The *p*-values for RMCNet and Unetr in the DSC and JCC metrics are much lower than 0.05, indicating significant differences between the two models in these metrics. In contrast, the *p*-values for the HD and ASD metrics are greater than 0.05, suggesting that the differences between the two models in these metrics are not significant, with both exhibiting similar feature recognition capabilities, although Unetr has a slight advantage in handling the boundary details of liver cancer. Despite RMCNet being slightly inferior to Unetr in the HD and ASD metrics, the overall performance of RMCNet, after integrating multiple modules, is excellent across all four metrics, showcasing its strong segmentation capability and comprehensive advantages.

To evaluate the generalization capability of the model, we conducted external testing of the proposed method on the publicly available 3Dircadb-01 dataset to verify its adaptability to different populations and imaging protocols. As shown in Table 4, RMCNet’s performance on various metrics was comparable to that on the test set, with a DSC of 72.96%, JCC of 71.25%, HD of 15.03 mm, and ASD of 10.21 mm on average. The proposed method exhibits a certain decline in performance on the external test set, which may be attributed to the difference in data source.

### 3.3. Discussion

In this part of the study, we analyze and discuss the segmentation performance of the proposed method on the test set. Considering the four metrics—DSC, JCC, HD, and ASD—RMCNet demonstrates the best liver tumor segmentation capability compared to other methods. However, in terms of the HD and ASD, RMCNet’s performance is slightly inferior to that of Unetr, which combines convolutional neural networks and Transformer architecture.

Considering the limitation of the article length, we randomly selected abdominal CT images of different scales and different shapes from the prediction results for display. According to the results of our statistical analysis of the dataset, it is shown that the volumes of each lesion in the dataset range from 1.4622 mm^3^ to 968,616.6677 mm^3^; the quartiles are 144.9219 mm^3^, 567.000 mm^3^, and 2768.0958 mm^3^; and the standard deviation is 52,981.1819 mm^3^. The lesions are divided into three categories of large-, medium-, and small-scale according to the size of the tumor volume. The lesion volumes counted are arranged in ascending order from small to large. We divided the tumors into three categories based on the tumor size tertiles: tumors with a volume less than 244.5200 mm^3^ were classified as small-scale tumors, those with a volume between 244.5200 mm^3^ and 1895.6548 mm^3^ were classified as medium-scale tumors, and those with a volume greater than 1895.6548 mm^3^ were classified as large-scale tumors.

**Figure 7:**
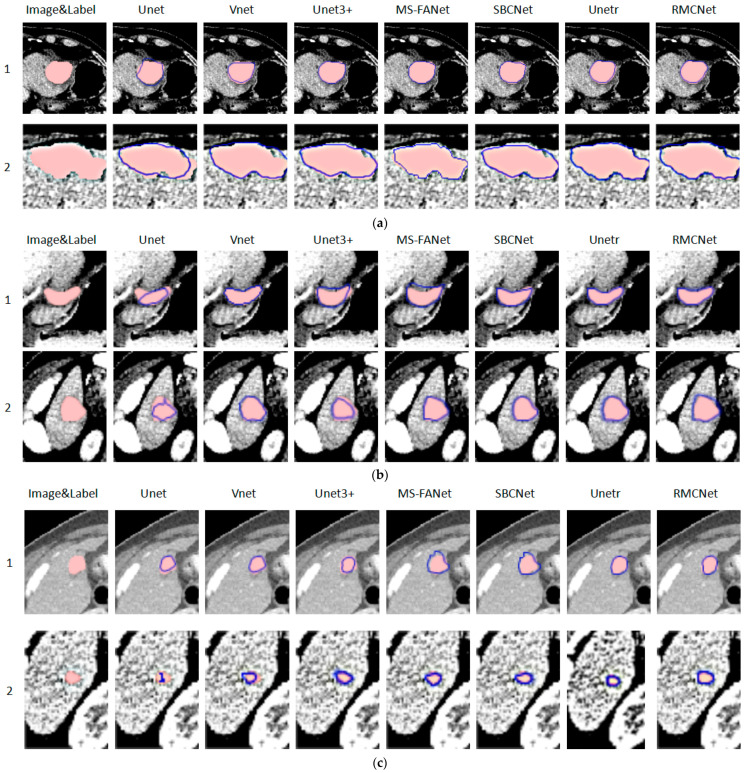
The segmentation results of different methods for liver tumors of different scales. (**a**) Different methods segment large-scale tumors. (**b**) Different methods segment medium-scale tumors. (**c**) Different methods segment small-scale tumors.

As shown in Figure 7, the volumes of the tumor lesions of concern in Figure 7a from top to bottom are 2803.3427 mm^3^ and 3892.7658 mm^3^, respectively, which are large-scale tumors; the volumes of the tumor lesions of concern in Figure 7b are 587.5528 mm^3^ and 863.4229 mm^3^, respectively, which are medium-scale tumors; and the volumes of the tumor lesions of concern in Figure 7c are 81.3142 mm^3^ and 49.3152 mm^3^, respectively, which are small-scale tumors. In each image, the red highlighted area represents the manually annotated liver tumor region, while the area enclosed by the blue contour represents the model’s predicted liver tumor region.

As shown in Figure 7, all methods can identify the specific locations of different liver cancer lesions in the CT images, but for the ability to recognize tumor boundaries of different scales, the ability of Unet to accurately locate the tumor boundaries is poor and cannot completely segment the tumor area. In contrast, other methods have better segmentation performance and can more accurately identify the boundaries for some tumors with special shapes. Although the segmentation performance of Vnet and Unet3+ is significantly better than that of Unet, over-segmentation still occurs. For the smaller-scale tumors in abdominal CT images, the Unet and Vnet models perform poorly, with serious under-segmentation of Unet, Vnet, and Unet3+, and they cannot accurately identify the edge information of the tumor. In comparison, RMCNet and Unetr significantly outperform other models in segmentation performance, excelling across various evaluation metrics. MS-FANet and SBCNet demonstrate moderate performance, with segmentation results at a mid-range level. By carefully comparing the segmentation results of RMCNet and Unetr for liver tumors of different sizes, we found that when segmenting larger and more complex tumors (as shown in Figure 7a, row 2, column 7 compared to column 8), both RMCNet and Unetr achieved high overlap with the ground truth, but Unetr demonstrated stronger boundary recognition. However, for the segmentation of smaller tumors (as shown in Figure 7c, row 2, column 7 compared to column 8), RMCNet exhibited superior segmentation capabilities, with higher overlap with the ground truth.

The Unetr model combines U-Net and the Transformer. Unlike traditional neural networks, it uses the Vision Transformer (ViT) as the encoder, which effectively captures global information and long-range dependencies in the input image through the self-attention mechanism. Therefore, Unetr is able to more accurately locate target boundaries when segmenting complex and irregularly shaped objects, reducing the issues of boundary blurring and loss of details. However, since the Transformer tends to focus on capturing global semantic information, its performance in handling small and less prominent local details may not be as strong as models based on more refined convolutional operations. In contrast, RMCNet is designed with careful consideration of the variations in the size, shape, and location of liver cancer. It introduces multi-scale convolutions, a convolutional attention mechanism, and residual paths in the encoding stage of the network. The convolutional attention mechanism helps the model focus on key features and highlight important regions. Multi-scale 3D convolutions, combined with convolutions of different receptive fields, allow the model to comprehensively extract features of the liver and tumor from various angles and scales. The 3 × 3 × 3 convolution kernel, with its smaller receptive field, is primarily used to capture fine local features, which is the main reason RMCNet outperforms Unetr in segmenting small tumors. Additionally, the residual path design helps preserve more local details during feature extraction, enhancing its performance in segmenting small- to medium-sized tumors. At the same time, the 5 × 5 × 5 convolution kernel, with its larger receptive field, can extract features that contain more contextual information, while the 7 × 7 × 7 convolution kernel enriches the feature representation from a more global perspective, covering larger region features and broader contextual information, which helps capture the overall shape of the tumor and larger-scale structural features. Although the 7 × 7 × 7 convolution assists in extracting certain global features, its receptive field is still limited. Therefore, Unetr performs better than RMCNet when dealing with large-scale liver cancer regions.

As shown in Table 5, the segmentation results of the various methods for large, medium, and small tumors also reflect the same trend. When segmenting tumors of three different sizes, except for Unetr’s superior performance on large-scale tumors, RMCNet outperformed other methods in the segmentation of medium-scale and small-scale tumors. Overall, RMCNet demonstrated better segmentation performance across tumors of varying sizes.

According to current results, we can draw a conclusion that compared with other methods, RMCNet shows the best segmentation performance. Most models can segment the large-area tumor areas in abdominal CT images relatively well. However, for some samples with smaller tumor scales and more complex shapes, the proposed network model has a better recognition ability and can better adapt to changes in different scales and shapes to achieve a complete segmentation of the tumor.

## 4. Conclusions

In this study, we designed a network, RMCNet, which is applied to the liver tumor segmentation of abdominal CT images based on attention, 3D multi-scale convolution and the residual path. The experimental results on the test set show that compared with other segmentation methods, this method has excellent qualitative and quantitative performance. The obtained results are closer to the real situation, further improving the accuracy of liver tumor segmentation. However, although this method has good performance, there are still certain limitations. In 3D multi-scale convolution, the receptive field of the 7 × 7 × 7 convolution kernel is limited. When processing global features, convolution operations confined to a local perspective may not effectively capture key features that are distant from the center, resulting in a lower accuracy of the model in large-scale tumor segmentation. Additionally, due to the lack of available clinical datasets, this experiment has not yet been validated on actual clinical data. Although the model has demonstrated a certain degree of generalization capability on the 3DIRCADb dataset, its performance is not ideal due to the differences in data distribution between the two datasets. In future research, we will focus more on improving the model’s segmentation performance for large-scale tumors and enhancing its generalization capability across datasets with different distributions.

## Figures and Tables

**Table 1:** Parameters and time complexity of the models.

Model	Params (M)	Training Time (s)	FLOPs (G)	Inference Time (s)
Unet	1.981	18.10	5.239	0.542
Vnet	45.598	79.42	213.633	5.864
Unet3+	22.403	90.82	251.056	7.067
MS-FANet	10.330	30.11	43.198	4.873
Unetr	92.618	60.25	55.024	1.412
SBCNet	9.140	41.77	39.716	4.460
RMCNet	5.192	21.45	20.397	0.728

**Table 2:** The segmentation performance of the model and other models on LiTS test dataset.

Model	DSC (%)	JCC (%)	HD (mm)	ASD (mm)
Unet	60.113 ± 1.08	63.780 ± 1.29	28.56 ± 14.58	8.16 ± 6.08
Vnet	66.563 ± 1.41	67.320 ± 1.45	47.48 ± 18.91	11.69 ± 11.85
Unet3+	65.014 ± 1.15	69.890 ± 1.32	40.79 ± 14.88	9.34 ± 6.10
MS-FANet	71.913 ± 0.61	72.184 ± 1.42	15.38 ± 6.47	5.27 ± 1.95
Unetr	74.151 ± 0.68	73.274 ± 0.54	11.03 ± 5.26	2.32 ± 1.02
SBCNet	73.225 ± 0.83	72.616 ± 0.53	13.75 ± 5.12	3.84 ± 1.55
RMCNet	76.566 ± 0.32	75.820 ± 0.4	11.07 ± 4.89	2.54 ± 1.78

**Table 3:** Statistical test analysis results.

Metric	DSC (%)	JCC (%)	HD (mm)	ASD (mm)
RMCNet and Unetr	1.91 × 10^−21^	4.03 × 10^−28^	0.973	0.553
RMCNet and Unet	1.10 × 10^−41^	1.51 × 10^−34^	2.31 × 10^−7^	1.91 × 10^−5^
RMCNet and Vnet	4.60 × 10^−29^	5.15 × 10^−27^	4.47 × 10^−12^	0.0001
RMCNet and Unet3+	5.56 × 10^−35^	1.77 × 10^−23^	1.22 × 10^−12^	8.70 × 10^−7^
RMCNet and MS-FANet	7.77 × 10^−36^	1.37 × 10^−15^	0.004	3.19 × 10^−7^
RMCNet and SBCNet	1.06 × 10^−22^	1.31 × 10^−33^	0.039	0.004

**Table 4:** The segmentation performance of the RMCNet on 3DIRCADb-01 dataset.

Model	DSC (%)	JCC (%)	HD (mm)	ASD (mm)
RMCNet	72.961 ± 0.47	71.246 ± 0.57	15.03 ± 4.47	10.21 ± 1.53

**Table 5:** Segmentation results of different models for tumors of different sizes.

Model	Small-Scale Tumors		Medium-Scale Tumors		Large-Scale Tumors	
	DSC (%)	JCC (%)	DSC (%)	JCC (%)	DSC (%)	JCC (%)
Unet	49.639 ± 0.33	50.65 ± 0.42	69.63 ± 0.32	73.83 ± 0.46	75.100 ± 0.34	77.86 ± 0.22
Vnet	51.870 ± 0.49	52.93 ± 0.37	73.151 ± 0.51	79.37 ± 0.45	86.617 ± 0.58	81,86 ± 0.47
Unet3+	51.221 ± 0.29	55.81 ± 0.33	81.525 ± 0.36	80.59 ± 0.41	84.293 ± 0.47	85.67 ± 0.46
MS-FANet	53.500 ± 0.25	54.30 ± 0.22	83.111 ± 0.32	82.60 ± 0.28	91.623 ± 0.18	89.95 ± 0.16
Unetr	54.481 ± 0.19	54.02 ± 0.15	84.170 ± 0.26	83.95 ± 0.29	95.802 ± 0.10	94.65 ± 0.09
SBCNet	56.340 ± 0.21	57.01 ± 0.19	83.764 ± 0.27	83.17 ± 0.24	92.711 ± 0.12	91.23 ± 0.11
RMCNet	61.491 ± 0.11	62.21 ± 0.09	84.880 ± 0.15	84.34 ± 0.13	95.330 ± 0.07	92.91 ± 0.07

## Data Availability

LiTS-Liver Tumor Segmentation Challenge Dataset is available at https://competitions.codalab.org/competitions/17094 (accessed on 7 March 2024). Liver segmentation 3D-IRCADb-01 Dataset is available at https://www.ircad.fr/research/data-sets/liver-segmentation-3d-ircadb-01 (accessed on 7 March 2024).
